# Identification of Chemical Composition of Leaves and Flowers from *Paeonia rockii* by UHPLC-Q-Exactive Orbitrap HRMS

**DOI:** 10.3390/molecules21070947

**Published:** 2016-07-21

**Authors:** Jinhua Li, Gang Kuang, Xiaohu Chen, Rui Zeng

**Affiliations:** 1 College of Pharmacy, Southwest University for Nationalities, Chengdu 610041, China; 15680690159@163.com; 2 Department of Biological and Chemical Engineering, Chongqing University of Education, Chongqing 400067, China; 1548094613dhj@sina.com; 3 Chongqing Institute for Food and Drug Control, Chongqing 401120, China; chenxiaohu78@126.com

**Keywords:** *Paeonia rockii*, UHPLC-Q-Exactive Orbitrap HRMS, chemical composition, high-resolution mass spectrometry

## Abstract

The Paeonia genus, an important source of crude drugs, has been extensively used in traditional Chinese medicine (TCM) to treat cardiovascular and female-related diseases. Although many peony species have been investigated, the study of *Paeonia rockii* is still quite limited, especially its chemical composition. Here, an advanced ultra-high-performance liquid chromatography (UHPLC) analytical technique combined with Q-Exactive Orbitrap hybrid quadrupole-Orbitrap mass spectrometry utilizing high-resolution full MS and MS/MS scan modes was applied to screen and identify the chemical constituents of this species. As a result, a total of 46 compounds were characterized, including 11 monoterpene glycosides, five phenolic acids, six tannins and 24 flavonoids. Among them, 16 compounds were reported for the first time in *Paeonia rockii*.

## 1. Introduction

The genus Paeonia, which includes 35 species [1], is distributed in Asia and the Mediterranean region and known for its ornamental value and medicinal properties [2]. In China, tree peonies, commonly called “King of Flowers” [3], have a long medicinal history due to their wide range of biological activities, such as analgesic, sedative, anti-inflammatory, antimicrobial, cardiovascular protection activities and so on. Many peony species have been applied to the treatment of cardiovascular and female diseases and their pharmacological effects and chemical constituents have been widely investigated [4,5]. *Paeonia rockii*, one of the most important species in Paeonia (section Moutan DC) [3], is a rare cold-resistant woody shrub which is distributed in the mountains of northwestern China, mainly north of Sichuan, south of Shaanxi and Gansu, west of Hubei and Henan as well as Tibet area, at an altitude of 1100–2800 m [6]. Although there is scant information on its medical use, the seed oil has been approved as a new food resource in China [7]. Up to now, few studies have systematically investigated the chemical composition of *Paeonia rockii*, particularly the leaves and flowers. Reviews on the genus only reported a few flavonoids identified in the flowers of *Paeonia rockii* and recent researches merely reported that the roots contain few compounds [2,4,5].

The Q-Exactive is a recent benchtop hybrid Orbitrap mass spectrometer combining the high resolving power (RP) performance of the Orbitrap with the selectivity of the quadrupole. This technology has slightly better performance than Orbitrap, with an RP of up to 140,000 FWHM at *m*/*z* 200 and a mass accuracy of less than 2 ppm. The MS/MS mode of targeted ion fragmentation (tMS2) and high-resolution full scan mode (FS) are applied in this instrument. The use of the tMS2 mode, which couples high-resolution and tandem mass spectrometry, achieves high sensitivity and selectivity [8]. Ultra-high-performance liquid chromatography (UHPLC) could be applied to rapidly detect more compounds in shorter running times and can be more easily coupled to MS than high-performance liquid chromatography (HPLC). Thus, UHPLC-Q-Exactive Orbitrap HRMS is suitable for the rapid identification of complex compound mixtures from plants. In the present work, the chemical compositions of flowers and leaves from *Paeonia rockii* were systematically investigated by UHPLC-Q-Exactive Orbitrap HRMS.

## 2. Results and Discussion

### 2.1. Identification of Chemical Compositions

**Figure 1:**
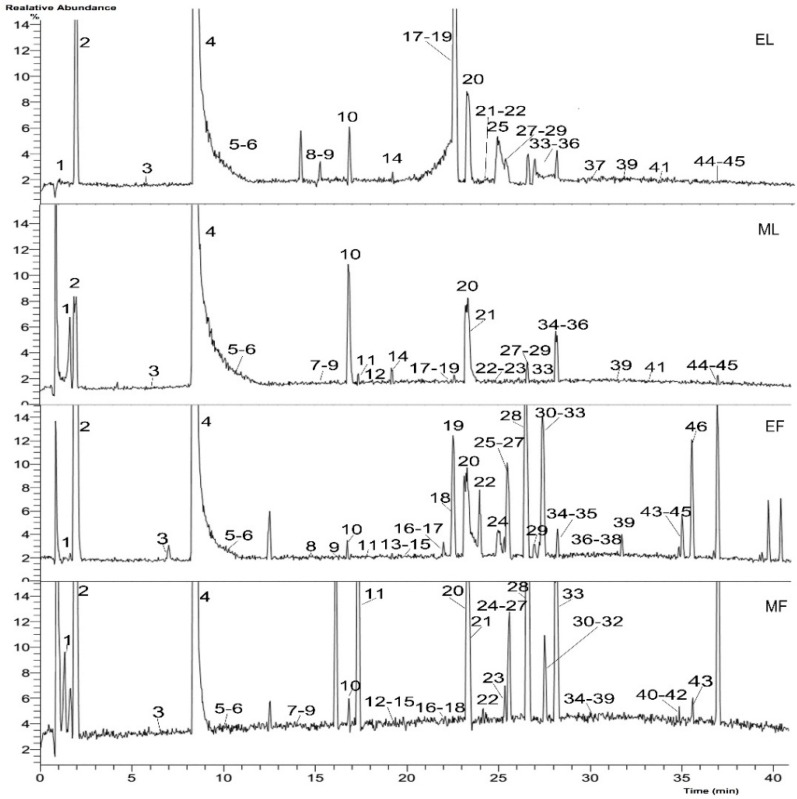
Base peak chromatogram (BPC) of the extracts from *Paeonia rockii* leaves and flowers (negative mode). EL, ethyl acetate fraction of the methanol extract of the leaves; ML, methanol extract of the leaves; EF, ethyl acetate fraction of the methanol extract of the flowers; MF, the methanol extract of the flowers.

**Figure 2:**
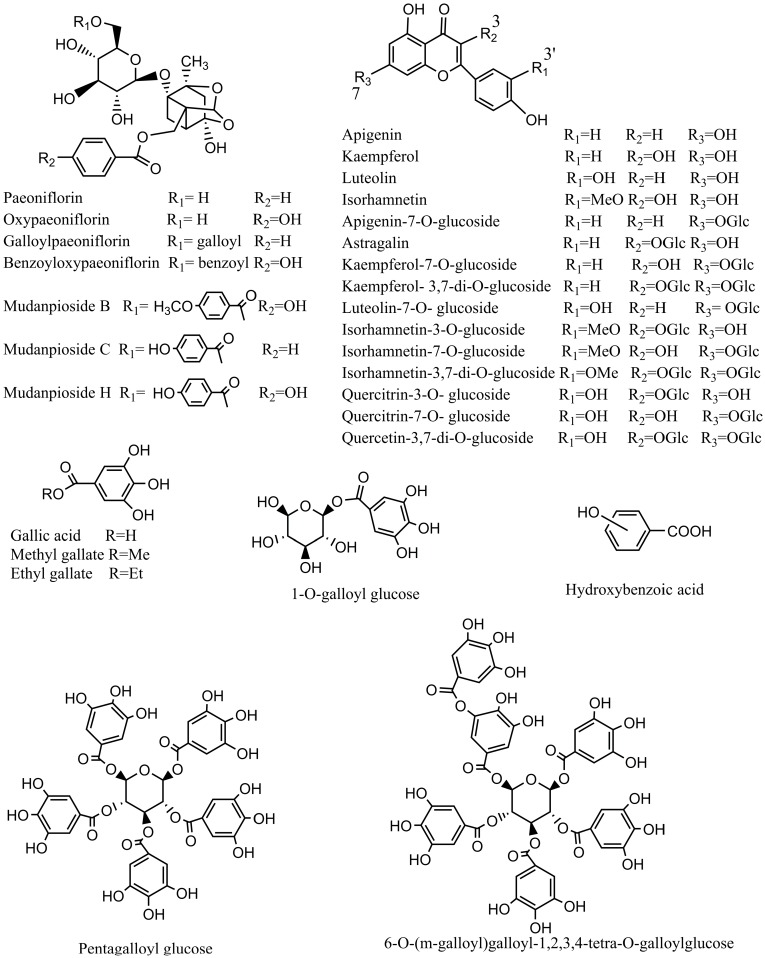
Chemical structures of the main identified compounds in the leaves and flowers from *Paeonia rockii*.

Identification of the chemical composition of the extracts of leaves and flowers was carried out by UHPLC-Q-Exactive. According to the retention time and the molecular ions, together with the major fragments that were observed in MS or MS^2^ spectra and followed by searching in online database (METLIN) and the literature, the chemical structures were tentatively identified and definitely classified into four groups: monoterpene glycosides, phenolic acids, tannins and flavonoids. The retention time (t_R_), molecular formula, exact mass, experimental mass, error, MS/MS data and identified compounds were presented in Table 1. Figure 1 and Figure 2 showed the base peak chromatogram (BPC) of the extracts and the chemical structures of identified compounds, respectively.

#### 2.1.1. Monoterpene Glycosides

Eleven monoterpene glycosides were detected in the extracts. Peaks 5, 13 and 15, both with *m*/*z* 495, showed the same fragment ions in the MS/MS spectrum. According to [9], peak 5 was characterized as oxypaeoniflorin, consistent with the fragmentation of the standard compound. Peaks 13 and 15 were characterized as oxypaeoniflorin isomers.

Peak 10, at *m*/*z* 479, was identified as paeoniflorin because it showed fragments at *m*/*z* 449, 327, 165 and 125. This fragmentation pattern was consistent with that of a standard compound, as well as reports in previous studies [9,10]. Peaks 24 and 31 also showed similar fragments and were proposed to be paeoniflorin isomers. Peak 18 was characterized as galloylpaeoniflorin and had a [M − H]^−^ at *m*/*z* 631; the fragments matched with literature reports [9,11].

Peak 26 had a molecular ion at *m*/*z* 615. In the MS/MS spectrum, this peak displayed fragment ions at *m*/*z* 585 [M − H − CH_2_O]^−^ and 447 [M − H − CH_2_O − (*p*-hydroxybenzoic acid)]^−^. Thus, compared to the literature data [9,11], peak 26 was characterized as mudanpioside H. Peaks 37 and 39, which presented the same molecular formula of C_30_H_32_O_13_ were identified as benzoyloxypaeoniflorin and mudanpioside C by comparison with a previous report [9], respectively. Peak 44 exhibited a [M − H]^−^ at *m*/*z* 629 and was identified as mudanpioside B. In the MS/MS spectrum, fragment ions 507, 479, 449, 165 and 121 were detected, in agreement with previous reports [9].

#### 2.1.2. Phenolic Acids

Based on MS, MS/MS data and the literatures, five phenolic acids were detected in the leaves and flowers. Gallic acid and its derivatives all showed fragments of *m*/*z* 169 and 125. The ion at *m*/*z* 169 was identified as the gallic acid molecular ion. The fragment ion at *m*/*z* 125 was related to the gallic acid loss of CO_2_ [12]. Peaks 2, 4 and 9 were proposed to be gallic acid [M − H]^−^ 169, methyl gallate [M − H]^−^ 183 and ethyl gallate [M − H]^−^ 197, respectively. In addition, methyl digallate, with the molecular ion at *m*/*z* 335, was also identified, showing fragment ions at *m*/*z* 183, 168 and 124 [13]. Peak 3 was proposed to be hydroxybenzoic acid with the molecular ion [M − H]^−^ 137, as reported in *Paeonia suffruticosa* [9]. The fragment at *m*/*z* 93 was identified as [M − H − CO_2_]^−^.

#### 2.1.3. Tannins

Six tannins were detected in the extracts. Peak 1 at *m*/*z* 331 was characterized as 1-*O*-galloylglucose because it showed fragments of *m*/*z* 169 [M − H − 162]^−^ and 125 [M − H − 162 − 44]^−^, corresponding to the loss of glucosyl and CO_2_. In addition, digalloyl glucose, trigalloyl glucose, tetragalloyl glucose, pentagalloyl glucose and 6-*O*-(*m*-galloyl)galloyl-1,2,3,4-tetra-galloylglucose were also identified and presented fragment ions *m*/*z* 169 and 125 in the MS/MS spectrum, in agreement with gallic acid. Peak 6, with the molecular ion at *m*/*z* 483, was identified as digalloyl glucose and also presented a fragment ion at *m*/*z* 331 in the base peak of the MS/MS spectrum, related to the possible loss of a galloyl. Peak 8 at *m*/*z* 635 was characterized as trigalloyl glucose because of the fragment ions at *m*/*z* 465, 313, 169 and 125. Peak 14 at *m*/*z* 787 was proposed to be tetragalloyl glucose due to the presence of fragments of *m*/*z* 617, 465, 313, 169 and 125. Peak 20, with the molecular ion at *m*/*z* 939, showed fragment ions at *m*/*z* 787, 635, 465, 313, 169 and 125 in the MS/MS spectrum. The fragmentation pattern is consistent with the previous reports [9,11]. Thus, peak 20 was tentatively identified as pentagalloyl glucose. Peak 29 had similar fragment ions as those of peak 20, except for a molecular ion at *m*/*z* 1091. Thus, based on fragment ions, peak 29 was proposed as 6-*O*-(*m*-galloyl)galloyl-1,2,3,4-tetra-galloylglucoside. Those tannins were detected in the leaves and flowers except for peak 20, which was only detected in the leaves.

#### 2.1.4. Flavonoids

Quercetin derivatives (peak 7, 16, 17 and 22) were identified. Peak 22, with the molecular ion *m*/*z* 463, was proposed to be quercetin-3-*O*-glucoside and showed fragments of *m*/*z* 301 [M − H − glucosyl]^−^, 300 [M − 2H − glucosyl]^−^ and 271 [M − H − glucosyl − CH_2_O]^−^ in the MS/MS spectrum. These MS^2^ data match the fragment ions of a standard compound. Peak 16 showed a similar fragmentation as that of quercetin-3-*O*-glucoside, and thus was identified as a quercetin-7-*O*-glucoside. Peak 17, with the molecular ion *m*/*z* 615, was proposed to be quercetin galloylglucoside because of the fragment ions *m*/*z* 463, 301, 300, and 169. Peak 7 presented fragment ions *m*/*z* 625, 462 and 301, related to the loss of glucosyl, and was proposed to be quercetin-3,7-diglucoside.

Peaks 41 and 43, both with the molecular ion 285 [M − H]^−^, were proposed to be luteolin and kaempferol, respectively, from a comparison with an online database (METLIN) and literature [14]. These peaks were also identified using standard compounds. Peaks 11, 25, 27, 32 and 33 were identified as kaempferol derivatives since they showed some similar fragmentation pattern to kaempferol. Peak 11 at *m*/*z* 609 was identified as kaempferol-3,7-di-*O*-glucoside because it showed a fragment of *m*/*z* 447 [M − H − 162]^−^, 446 [M − 2H − 162]^−^, 285 [M − H − 162 − 162]^−^ and 283 [M − 3H − 162 − 162]^−^. Peaks 25 and 33, both at *m*/*z* 447, when compared to an online database (METLIN), were identified to be kaempferol-7-*O*-glucoside and astragalin, respectively. Peak 27 at *m*/*z* 599 was identified as kaempferol galloylglucoside with fragments of *m*/*z* 447 [M − H − 152]^−^ and 285 [M − H − 162]^−^ after the loss of galloyl and glucosyl, respectively. Peak 32 at *m*/*z* 593 was identified as kaempferol glucosylrhamnoside because of the fragments at *m*/*z* 431 [M − H − 162]^−^ and 285 [M − H − 162 − 146]^−^. Peak 21 was identified as luteolin-7-*O*-glucoside based on comparison to a standard compound.

Peaks 42 and 46 both showed the molecular ion [M − H]^−^ *m*/*z* 315, and their fragmentation patterns were similar, with only differences between the relative intensities of the peaks. Compared to a standard compound, peak 46 was identified as isorhamnetin. Peak 42 was identified as an isorhamnetin isomer. Peak 12 was identified as isorhamnetin 3,7-*O*-glucoside and had fragments of *m*/*z* 476 [M − 2H − 162]^−^, 315 [M − H − 162 − 162]^−^ and 314 [M − 2H − 162 − 162]^−^, in agreement with a previous report [9]. Peaks 30 and 35 showed the same molecular ions at *m*/*z* 477, identified to be isorhamnetin-7-*O*-glucoside and isorhamnetin-3-*O*-glucoside, respectively. They both with a fragment ion at *m*/*z* 315 after the loss of a glucosyl.

Peak 23 at *m*/*z* 593 was identified as apigenin diglucoside due to the fragment ions at *m*/*z* 431 [M − H − 162]^−^ and 269 [M − H − 162 − 162]^−^. Peak 28 at *m*/*z* 431 was identified as apigenin glucoside because of the fragment at 431 [M − H − 162]^−^. Peak 34 at *m*/*z* 577 was identified as apigenin rhamnoglucoside and exhibited the fragments at *m*/*z* 431 [M − H − 146]^−^ and 269 [M − H − 146 − 162]^−^, which are related to the loss of rhamnosyl and glucosyl, respectively. Peaks 36, 38 and 40, both at *m*/*z* 583, presented fragment ions at *m*/*z* 431, 269 and 169. Thus, these peaks were identified as apigenin galloylglucoside. Due to the limitation of MS information, exact groups position was not sure. Peak 45 at *m*/*z* 269 was identified as apigenin using a standard compound. Its fragmentation was also consistent with previous reports [14].

A total of 46 compounds were tentatively identified, including 11 monoterpene glycosides, five phenolic acids, six tannins and 24 flavonoids. Some isomers, like oxypaeoniflorin, paeoniflorin and apigenin galloylglucoside, were detected in this work because the MS data did not provide enough information to establish the exact positions of the substituent groups. Sixteen compounds, including digalloyl glucose, methyl digallate, trigalloyl glucose, isorhamnetin-3-*O*-glucoside, isorhamnetin7-*O*-glucoside, apigenin-7-*O*-glucoside, apigenin galloylglucoside, kaempferol-7-*O*-glucoside, kaempferol-3,7-di-*O*-glucoside, astragalin, quercetin-3,7-di-*O*-glucoside, quercetin-3-*O*-glucoside, isorhamnetin-3,7-di-*O*-glucoside, quercetin galloylglucoside, luteolin-7-*O*-glucoside and kaempferol galloylglucoside, were reported for the first time in the *Paeonia rockii*. Compared with other peony species, such as *Paeonia suffruticosa* and *Paeonia lactiflora*, we found some of the same compounds, such as paeoniflorin, oxypaeoniflorin, benzoyloxypaeoniflorin, luteolin, apigenin, kaempferol [15,16]. However, triterpenes, steroids, resveratrol trimers, paeonol and its derivatives [4,5], generally contained in seeds and roots of the Paeonia genus, were not detected in our work. This indicates that there is variation on chemical compositions of different parts and different peony species. Interestingly, methyl gallate, one of the most important pharmaceutical intermediates, was unexpectedly abundant in the leaves and flowers based on the chromatograms (Figure 1, Peak 4) and far more so than other identified compounds, which shows that the leaves and flowers of *P. rockii* could serve as a promising source of methyl gallate. To sum up, UHPLC-Q-Exactive Orbitrap HRMS is a fast and reliable technique for the identification of complex chemical components in herbs and this study provides the basic phytochemical information for further research and exploitation of *Paeonia rockii*.

## 3. Material and Methods

### 3.1. Chemicals

The following chemicals were purchased from Chengdu Must Biotechnology Co., Ltd. (Sichuan, China): paeoniflorin, oxypaeoniflorin, luteolin-3-*O*-glucoside, quercetin-3-*O*-glucoside, luteolin, kaempferol and isorhamnetin. AR-grade reagents that were used for plant extraction were obtained from Chengdu Kelong Chemical Co., Ltd. (Sichuan, China). HPLC-grade acetonitrile (Sigma-Aldrich, St. Louis, MO, USA) and de-ionized water were purified using a Milli-Q system (Millipore, Bedford, MA, USA) and used for UHPLC-Q-Exactive analysis.

### 3.2. Plant Material Collection and Sample Preparation

Samples of *Paeonia rockii* were collected Songpan, located in northwestern Sichuan, China, in May 2014. The plant material comprised individually separated leaves and flowers. The sample leaves and flowers were preserved in the Herbarium of the Southwest University for Nationalities (SWUN). The leaves and flowers were frozen at −20 °C. The samples were ground to a fine powder in a mechanic grinder with a 65 mesh size. Then, 10 g of plant material was extracted three times with 100 mL of 100% methanol using ultrasound-assisted solvent extraction for 1 h at room temperature. The filtered extracted solutions were concentrated using a rotary evaporator. Some of the dry methanol extracts were resuspended in pure water and successively extracted with petroleum ether followed by ethyl acetate (10 times) using the liquid-liquid extraction method, and the solvent was removed using a rotary evaporator to obtain the dry form of each fraction. The methanol extracts and the ethyl acetate fractions of the methanol extracts of plant materials were dissolved in 100% methanol to used for UHPLC-Q-Exactive analysis. The extracts had concentrations (*m*/*v*) of 5 mg/mL and were filtered through 0.22 μm Nylon micropore membranes prior to injection.

### 3.3. UHPLC-Q-Exactive Analysis

#### 3.3.1. Liquid Chromatography

UHPLC analyses were performed using an Ultimate 3000 (Dionex, Sunnyvale, CA, USA) system that was equipped with an online vacuum degasser, a quaternary pump, an autosampler and a thermostatted column compartment. The column that was used for the chromatographic separation was an ACQUITY UPLC BEH C18 2.1 mm × 100 mm, 1.7 μm (Waters, Milford, MA, USA) at 35 °C. The separation conditions consisted of a gradient elution using aqueous formic acid 0.1% (*v*/*v*) as mobile phase A and acetonitrile as phase B at a flow rate of 0.3 mL/min. The following gradient was applied: 0–2 min, 5% B; 2–10 min, 5%–15% B; 10–30 min, 15%–40% B; and 30–40 min, 40%–60% B. The injection volume was 2 μL and the injection temperature was 15 °C.

#### 3.3.2. Mass Spectrometry

Tandem mass spectrometry was performed with a Q Exactive Orbitrap MS (Thermo Fisher, Waltham, MA, USA) using a heated electrospray ionization source for the ionization of the target compounds in the negative ion mode. The operating parameters were as follows: spray voltage, 2.00 KV; sheath gas pressure, 30 psi; auxiliary gas pressure, 10 arb; capillary temp, 320 °C; auxiliary gas heater temp, 350 °C; scan modes, full MS (resolution 70,000) and MS/MS (resolution 17,500, normalized collision energy 35 eV, stepped normalized collision energy 30 and 40 eV) and scan range, *m*/*z* 80–1200.

## 4. Conclusions

In summary, a novel UHPLC-Q-Exactive method was developed in the present work to successfully elucidate the complex chemical composition of *Paeonia rockii* leaves and flowers. A total of 46 compounds were characterized, including 11 monoterpene glycosides, five phenolic acids, six tannins and 24 flavonoids. Sixteen of these compounds were reported in *Paeonia rockii* for the first time. This study indicates that UHPLC-Q-Exactive Orbitrap HRMS is a fast and reliable technique to investigate complicated mixtures of plant components, and provides scientific evidence for future study and utilization of *Paeonia rockii*.

## Figures and Tables

**Table 1:** Characterization of chemical compound of extracts of leaves and flowers from *Paeonia rockii* by UHPLC-Q-Exactive in negative mode.

Peak	T_R_ (min)	Formula	*m*/*z* Calculated	*m*/*z* Experimental	Error (ppm)	MS/MS Fragments	Proposed Compound	Sample
1	1.55	C_13_H_16_O_10_	331.06597	331.06766	5.095	169, 125	1-*O*-Galloyl glucose	EF, EL, MF, ML
2	2.00	C_7_H_6_O_5_	169.01315	169.01328	0.771	125, 97	Gallic acid	EF, EL, MF, ML
3	6.99	C_7_H_6_O_3_	137.02332	137.02327	−0.37	93	Hydroxybenzoic acid	EF, EL, MF, ML
4	8.98	C_8_H_8_O_5_	183.0288	183.02907	1.476	168, 124	Methyl gallate	EF, EL, MF, ML
5	11.14	C_23_H_28_O_12_	495.1497	495.15182	4.276	465, 345, 281, 195, 165, 137	Oxypaeoniflorin ^a^	EF, EL, MF, ML
6	11.25	C_20_H_20_O_14_	483.07693	483.07922	2.288	331, 313, 169, 125	Digalloyl glucose	EL, ML
7	14.72	C_27_H_30_O_17_	625.13993	625.14282	2.894	462, 301, 299	Quercetin-3,7-diglucoside	MF, ML
8	14.99	C_27_H_24_O_18_	635.08789	635.09076	2.879	465, 313, 169, 125	Trigalloyl glucose	EF, EL, MF, ML
9	15.15	C_9_H_10_O_5_	197.04445	197.04492	2.386	169, 152, 125	Ethyl gallate	EF, EL, MF, ML
10	16.83	C_23_H_28_O_11_	479.15479	479.15692	4.449	327, 195, 165, 121	Paeoniflorin ^a^	EF, EL, MF, ML
11	17.15	C_27_H_30_O_16_	609.14501	609.14795	4.825	447, 446, 285, 283	Kaempferol-3,7-di-*O*-glucoside	EF, EL, MF, ML
12	18.22	C_28_H_32_O_17_	639.15558	639.15863	3.054	476, 315, 313	Isorhamnetin-3,7-di-*O*-glucoside	MF, ML
13	19.00	C_23_H_28_O_12_	495.1497	495.15189	4.418	465, 345, 281, 195, 151, 137	Oxypaeoniflorin isomer	EF, MF,
14	19.20	C_34_H_28_O_22_	787.09885	787.10205	4.067	617, 465, 313, 169, 125	Tetragalloylglucose	EF, EL, MF, ML
15	20.65	C_23_H_28_O_12_	495.1497	495.15195	4.539	465, 345, 281, 195, 151, 137	Oxypaeoniflorin isomer	EF, MF
16	21.61	C_21_H_20_O_12_	463.08710	463.08923	4.594	301, 257, 151	Quercetin-7-*O*-glucoside	EF, MF
17	21.89	C_28_H_24_O_16_	615.09806	615.10052	3.998	463, 301, 169, 151, 125	Quercetin galloylglucoside	EF, EL, MF, ML
18	22.29	C_30_H_32_O_15_	631.16575	631.16864	4.584	613, 491, 479, 399, 313, 271, 211, 169, 125, 121	Galloylpaeoniflorin	EF, EL, MF, ML
19	22.50	C_15_H_12_O_9_	335.03976	335.04150	5.198	183, 168, 124	Methyl digallate	EF, EL, ML
20	23.15	C_41_H_32_O_26_	939.10981	939.11316	3.570	787, 635, 617, 465, 447, 313, 295, 169, 125	Pentagalloyl glucose	EF, EL, MF, ML
21	23.60	C_21_H_20_O_11_	447.09219	447.0943	4.724	285	Luteolin-7-*O*-glucoside ^a^	EL, MF, ML
22	24.10	C_21_H_20_O_12_	463.08710	463.08914	4.400	301, 300, 271, 255, 179, 151	Quercetin-3-*O*-glucoside ^a^	EF, EL, MF, ML
23	24.95	C_27_H_30_O_15_	593.15010	593.15308	2.984	431, 269	Apigenin diglucoside	MF, ML
24	25.31	C_23_H_28_O_11_	479.15479	479.15707	4.637	449, 327, 195, 183, 151, 139, 121	Paeoniflorin isomer	EF, MF,
25	25.47	C_21_H_20_O_11_	447.09219	447.09412	4.322	285, 284, 257, 151	Kaempferol-7-*O*-glucoside	EF, EL, MF
26	25.56	C_30_H_32_O_14_	615.17083	615.17334	4.007	585, 477, 447, 431, 281, 239, 179, 137, 93	Mudanpioside H	EF, MF
27	25.65	C_28_H_24_O_15_	599.10315	599.10571	4.279	447, 313, 285, 284, 169, 151, 125	Kaempferol galloylglucoside	EF, EL, MF, ML
28	26.52	C_21_H_20_O_10_	431.09727	431.09882	3.588	269, 268	Apigenin-7-*O*-glucoside	EF, EL, MF, ML
29	26.93	C_48_H_34_O_30_	1091.12077	1091.12451	3.432	939, 787, 769, 635, 617, 465, 447, 431, 295, 169, 125, 123	6-*O*-(*m*-Galloyl)galloyl-1,2,3,4-tetragalloylglucose	EL, ML
30	27.04	C_22_H_22_O_12_	477.10275	477.10483	2.078	357, 315, 299, 287, 271, 169, 151	Isorhamnetin7-*O*-glucoside	EF, MF
31	27.22	C_23_H_28_O_11_	479.15479	479.15688	4.324	449, 327, 195, 183, 151, 139, 121	Paeoniflorin isomer	EF, MF
32	27.30	C_27_H_30_O_15_	593.15010	593.25183	2.734	431, 285	Kaepferol glucosyl rhamnoside	MF
33	27.40	C_21_H_20_O_11_	447.09219	447.09396	3.964	285, 284, 255, 227, 179, 151	Astragalin	EF, EL, MF, ML
34	28.10	C_27_H_30_O_14_	577.15518	577.15741	3.861	431, 413, 269	Apigenin rhamnoglucoside	EF, EL, MF, ML
35	28.19	C_22_H_22_O_12_	477.10275	477.10468	4.040	357, 314, 285, 271, 257, 243, 151	Isorhamnetin-3-*O*-glucoside	EF, EL, MF, ML
36	28.80	C_28_H_24_O_14_	583.10823	583.11066	2.428	431, 313, 269, 169, 125	Apigenin galloylglucoside isomer	EF, EL, MF, ML
37	30.36	C_30_H_32_O_13_	599.17592	599.17865	4.561	477, 447, 431, 285, 281, 239, 179, 169, 149, 137, 121, 93	Benzoyloxypaeoniflorin	EF, MF
38	31.06	C_28_H_24_O_14_	583.10823	583.11072	2.488	431, 313, 269, 169, 125	Apigenin galloylglucoside isomer	EF, MF
39	31.76	C_30_H_32_O_13_	599.17592	599.17847	4.260	477, 447, 195,165, 137, 121, 93	Mudanpioside C	EF, EL, MF, ML
40	32.82	C_28_H_24_O_14_	583.10823	583.11108	2.848	432, 431, 269, 268, 169, 125	Apigenin galloylglucoside isomer	EF, MF
41	32.89	C_15_H_10_O_6_	285.03936	285.04095	5.562	257, 175, 151, 133	Luteolin ^a^	EF, EL, MF, ML
42	33.64	C_16_H_12_O_7_	315.04993	315.05185	6.097	283, 255, 227, 211	Isorhamnetin isomer	EF, MF
43	35.00	C_15_H_10_O_6_	285.03936	285.04095	5.562	257, 229, 151	Kaempferol ^a^	EF, MF
44	35.40	C_31_H_34_O_14_	629.18648	629.18927	2.788	449, 347, 165, 121	Mudanpioside B	EF, EL, MF, ML
45	35.52	C_15_H_10_O_5_	269.04445	269.04602	5.836	225, 159, 151, 117, 107	Apigenin ^a^	EF, EL, MF, ML
46	35.88	C_16_H_12_O_7_	315.04993	315.18200	6.002	300, 271, 151	Isorhamnetin ^a^	EF

MF, the methanol extract of the flowers; ML, the methanol extract of the leaves; EF, ethyl acetate fraction of the methanol extract of the flowers; EL, ethyl acetate fraction of the methanol extract of the leaves. ^a^ Compared with a reference.
